# Targeted Suppression of the Tomato Pathogen *Alternaria alternata* via Exogenous Application of Double-Stranded RNA

**DOI:** 10.3390/jof12050373

**Published:** 2026-05-18

**Authors:** Andrey R. Suprun, Stanislava A. Vinogradova, Alina A. Beresh, Natalia S. Chopenko, Alina A. Dneprovskaya, Evgeniya V. Trubetskaya, Artem Yu. Manyakhin, Konstantin V. Kiselev

**Affiliations:** 1 Federal Scientific Center of the East Asia Terrestrial Biodiversity, Far Eastern Branch of the Russian Academy of Sciences, 690022 Vladivostok, Russia; 2 Institute of the World Ocean, Far Eastern Federal University (FEFU), 690922 Vladivostok, Russia

**Keywords:** RNA interference, dsRNA, *Alternaria alternata*, tomato, early blight, SIGS, plant protection, fungicide

## Abstract

Alternaria blight, caused by fungi of the genus *Alternaria*, is one of the most common and damaging diseases affecting tomatoes, leading to significant yield losses. The intensive use of chemical fungicides faces the problems of pathogen resistance development and negative environmental impacts. This study investigated the possibility of using RNA interference technology based on exogenous double-stranded RNAs (dsRNAs) to protect tomatoes against the causal agent of early blight (EB), *Alternaria alternata*. Key genes of the pathogen *A. alternata* were selected as targets: *Alt-a1* (a major allergen and virulence factor), *TEF1a* (translation elongation factor 1-alpha) and *β-Tub* (β-tubulin). Specific dsRNAs were synthesized in vitro and applied to tomato plants (*Solanum lycopersicum* L. cv. Micro-Tom) simultaneously with inoculation of *A. alternata* strain C7.24-T2-L-F1 spores. Visual assessment, measurement of chlorophyll A and B, and real-time quantitative PCR analysis showed that treatment with dsRNAs targeting the *Alt-a1*, *TEF1a* and *β-Tub* genes significantly suppressed infection development, reducing the amount of pathogen DNA in plant tissues by 7 to 27 times depending on the dsRNA type. The most effective was dsRNA to the *Alt-a1* gene. Thus, the obtained results demonstrate the promise of spray-induced gene silencing (SIGS) as a strategy for protecting tomato plants against the pathogen *A. alternata*.

## 1. Introduction

The tomato (*Solanum lycopersicum* L.) is one of the most widely cultivated and consumed vegetable crops in the world [1]. Phytopathogenic fungi pose is a serious threat to global food security. Among them, representatives of the genus *Alternaria* rank as one of the most harmful, causing early blight (EB) in tomato plants, characterized by necrotic lesions on leaves, stems, and fruits, leading to yield losses of up to 80% [2,3]. This pathogen is most dangerous in regions with high humidity and moderately warm climates, where prolonged night-time dew promotes disease development [4]. A high degree of infection leads to defoliation and significant economic losses within a short period.

Fungi of the genus *Alternaria* exhibit high ecological plasticity, acting as saprotrophs, endophytes, or aggressive pathogens depending on environmental conditions and genotypic characteristics [4,5]. Their ability to colonize a wide range of substrates—from plant tissues and agricultural products to soil—makes them a focus of intense research across various fields, including plant pathology, biotechnology, and ecology [6].

Controlling tomato EB has traditionally been based on the use of chemical fungicides. However, this strategy is associated with the risks of pathogen resistance development, the accumulation of toxic compounds in produce, and negative impacts on agroecosystems [7]. Regular applications at 7–10 day intervals do not always ensure effective control due to the high susceptibility of the crop and the prolonged persistence of conditions favorable for infection [4]. Furthermore, cases of *Alternaria* spp. resistance to fungicides have been reported, exacerbating the problem [8]. All of this highlights the need to search for alternative, environmentally safe plant protection methods [9].

A promising approach for combating tomato EB is Spray-Induced Gene Silencing (SIGS), based on the application of exogenous double-stranded RNAs (dsRNAs) to the surface of plants, followed by their penetration into plant and fungal tissues [10,11]. Once taken up by the pathogen’s cells, the dsRNA triggers the RNA interference (RNAi) mechanism. The dsRNA binds to the ribonuclease DCL, which cleaves it into small fragments, 20–25 nucleotides in length, each with two unpaired bases at the 5′ and 3′ ends. These fragments interact with the RISC complex, which cleaves one of the complementary RNA strands [12]. The resulting complex moves through the cell in search of homologous messenger RNA. Upon finding it, the AGO protein from the RISC complex cleaves the messenger RNA [10]. The specific degradation of complementary messenger RNAs leads to the suppression of vital gene expression and inhibition of infection development [13]. The high specificity of dsRNA action minimizes impact on the host plant and non-target organisms.

The effectiveness of the SIGS approach has been demonstrated in relation to protecting various crops from phytopathogenic fungi. It has been previously reported that treatment with dsRNAs targeting the *CYP51* gene of *Fusarium graminearum* and *Botrytis cinerea* suppressed infection on Arabidopsis, barley, and grapevine [14,15,16]. Treatment of tomato, apple, and grape plants with dsRNAs targeting the *VPS51*, *DCTN1*, *SAC1*, and *pgxB* genes of *Aspergillus niger* inhibited pathogen growth and symptom development, leading to a significant reduction in lesion size compared to the control [14]. Spraying with dsRNAs against the *Hsp90*, *EF-1α*, and *SDH* genes of *Phytophthora infestans* reduced growth, sporulation, and disease development in potato [17]. However, to date, there is limited data on the use of SIGS technology on tomato plants.

In this study, we report the successful use of exogenous synthetic dsRNAs complementary to the *Alt-a1*, *EF-1α*, and *β-Tub* genes of the fungus *A. alternata*. The experiments were conducted by analyzing the expression levels of target genes, assessing the severity of early blight symptoms, and evaluating the level of *A. alternata* DNA amplification in tomato leaves. It was shown that the application of dsRNA led to a reduction in leaf lesion area and a significant decrease in pathogen load within plant tissues.

## 2. Materials and Methods

### 2.1. Fungal Pathogen and Plant Inoculation

*Alternaria alternata* (Fr.) (strain C7.24-T2-L-F1) was isolated from naturally infected tomato leaves (cv. Dessert) with early blight (EB) symptoms collected from an experimental field in the Far Eastern Federal District of Russia (longitude 43.97641661616043 and latitude 132.4808241135788) in 2024. Isolates were grown on potato dextrose agar (PDA) in 9 cm glass Petri dishes at 25 °C in a climate chamber for 7 days. The resulting pure culture was identified to the species level based on the morphological and molecular genetic markers *ITS*, *RPB2*, *TEF1*, and *Alt-a1*. The sequences of the primers used are presented in Table S1. The resulting nucleotide sequences showed 100% homology to *A. alternata*.

Tomato plant (*Solanum lycopersicum* L.) cultivar Micro-Tom was grown under controlled conditions at 25 °C, with a 16 h photoperiod, and a light intensity of ~120 μmol m^−2^ s^−1^ in a chamber (Sanyo MLR-352, Panasonic, Osaka, Japan). The seed material (obtained within the framework of the state assignment of the Ministry of Science and Higher Education of the Russian Federation (subject number 124012200181-4)) was provided by the Laboratory of Biotechnology of the Federal Scientific Center for Terrestrial Biodiversity of East Asia, Vladivostok, Russia.

*A. alternata* spore suspension was prepared from 7-day-old actively growing cultures by adding 5 mL of 0.01% *v*/*v* Tween 20 to a Petri dish, after which the spores were washed off by pipetting. Spore numbers were measured using a Goryaev chamber and diluted to a concentration of 2 × 10^5^ spores mL^−1^. Leaves of 8-week-old tomato were droplet inoculated with 1 mL of the spore suspension at a concentration of 2 × 10^5^ spores/mL. One hour after inoculation, when the applied droplets with spores had dried, the plants were treated with 100 μg of dsRNA dissolved in 1 mL of water specific for regions of the *Alt-a1* (Alt-a1-dsRNA), *TEF1a* (TEF1a-dsRNA), and *β-Tubulin* (Tub2-dsRNA) gene transcripts of *A. alternata*. Spore inoculation without added dsRNA (Sp) served as a negative control, as did treatment with dsRNA complementary to the neomycin phosphotransferase II gene (NPTII-dsRNA), which is absent from the Alternaria and tomato genomes. For each dsRNA treatment, 100 μg of dsRNA was dissolved in 1 mL of nuclease-free water and then applied to tomato leaves. And the positive control was set by using 1 mL of a 0.2% solution of KS-carbendazim 500 g/L (Ferazim, AgroExpertGroup, Moscow, Russia), a well-known agent for controlling fungal plant pathogens that inhibits β-tubulin.

Three plants were used in a separate experiment for each treatment type. A total of three independent experiments were conducted. After inoculation, plants were incubated at 30 °C, 80–90% humidity, and a 16 h light cycle to stimulate EB development. Efficacy was assessed by the degree of suppression of necrotic symptoms caused by the *A. alternata* fungus on day 7 post-inoculation in Fiji software version 1.54p using the Trainable Weka Segmentation plugin [18]. This tool performs pixel-by-pixel image segmentation, combining image processing methods with machine learning algorithms. This allows for precise segmentation of diseased tissue from healthy tissue. The results are presented as a percentage, representing the ratio of the affected tissue area to the healthy tissue area.

### 2.2. Construction of Tub2, TEF1a and Alt-a1 Templates and Synthesis of dsRNAs

A large fragment of the targeted genes beta-tubulin (*Tub2*) (GenBank KY814630.1; 554 bp), translation elongation factor 1-alpha (*TEF1a*) (GenBank MN258358.1; 424 bp), and major allergen (*Alt-a1*) (GenBank MW387003.1; 512 bp) were amplified by RT-PCR using RNA samples isolated from *A. alternata* mycelium (strain C7.24-T2-L-F1). The primers are listed in Table S1. The RT-PCRs were performed in a Bis-M1105 Thermal Cycler (Bis-N, Novosibirsk, Russia). The RT-PCR products were subcloned into pJET1.2/blunt. The T7 promoter sequence was then introduced into the 5′ and 3′ ends of the *Tub2*, *TEF1a*, and *Alt-a1* genes by PCR, using pJET1.2/blunt plasmids containing the target gene sequences as a template. The PCR program was as follows: a denaturation step for 2 min at 95 °C, then 5 cycles of 10 s at 95 °C, 10 s at 65 °C, 38 s at 72 °C, followed by 35 cycles of 10 s at 95 °C, 48 s at 72 °C, and a final step of 2 min at 72 °C. Tub2-, TEF1a-, and Alt-a1-dsRNA were then synthesized using the in vitro T7 transcription kit (Biolabmix, Novosibirsk, Russia) according to the manufacturer’s protocol and using primers containing the T7 promoter sequence at the 5′ end (Table S1). The resulting dsRNA products were analyzed by gel electrophoresis in 2% *w*/*v* agarose gels and spectrophotometrically (NanoPhotometer P330, Implen, Munich, Germany) to assess purity, integrity, and quantity.

### 2.3. Evaluation of the In Vitro Effect of dsRNAs on the Growth of A. alternata

The effect of dsRNAs targeting the *Tub2*, *TEF1a*, and *Alt-a1* genes, as well as non-specific dsRNA targeting the *NPTII* gene, on the growth of *A. alternata* was tested in vitro using Petri dishes with potato dextrose agar (PDA) (HiMedia Laboratories, Thane, India). Samples (5 mm in diameter) were obtained from an actively growing colony of *A. alternata*. Each explant was placed in the center of a Petri dish, and then 100 µg (30 µL) of the respective dsRNA was immediately applied directly onto it for each treatment variant. Sterile water (30 µL) was used as a negative control. Additionally, the 0.2% solution of Ferazim (1 µL/mL) was used as a positive control. Growth dynamics were assessed by measuring the colony diameter at 1, 3, and 5 days post-dsRNA application. A total of six treatments were evaluated: (i) water (negative control); (ii) dsRNA targeting the *Alt-a1* gene (Alt-a1-dsRNA); (iii) dsRNA targeting the *TEF1a* gene (EF1-dsRNA); (iv) dsRNA targeting the *β-Tub* gene (Tub-dsRNA); (v) non-specific dsRNA targeting the *NPTII* gene (NPTII-dsRNA); (vi) 0.2% solution of KS-carbendazim 500 g/L (positive control).

### 2.4. DNA/RNA Isolation and Reverse Transcription Reaction

DNA was isolated from 50 mg of tomato leaves according to the method described by Echt et al. in 1992 [19], with some modifications. Briefly, 800 μL of Echta’s buffer was added to a test tube and placed in a thermostat at 60 °C. The tissue was placed in a mortar and ground with a pestle with the addition of 800 μL of the incubated Echta’s buffer; then, the contents were transferred to 1.5 mL test tubes. The tubes were incubated for 1–1.5 h in a thermostat at 60 °C, stirring regularly. After incubation, 300 μL of chloroform were added and mixed for 5 min. Then, the tube was centrifuged for 5 min at 13,000× *g* and 420 μL of the supernatant was collected in a new tube. 950 μL of ethyl alcohol was added and the tube was incubated at −20 °C for 20 min. Then, it was centrifuged for 7 min at 13,000× *g*. The alcohol was decanted and the DNA precipitate was dried for 15–20 min. The dried precipitate was dissolved in 100–150 µL of water. DNA concentration was measured using a NanoPhotometer P330 spectrophotometer (Implen, Munich, Germany).

For RNA extraction, diseased tomato leaves were collected seven days after inoculation. Total RNA was extracted as described previously [20]. cDNAs were synthesized using the MMLV RT Kit (Eurogen, Moscow, Russia). Reactions were carried out in a 40 μL reaction mixture that included first-strand buffer, 4 μL dNTP mix (10 mM each), 1.5 μL oligo-(dT)15 primer (100 μM), 4 μL DTT (dithiothreitol, 20 mM), and 3.4 μL MMLV reverse transcriptase (100 U/μL) at 37 °C for 80 min. The resulting products were then PCR-amplified and tested using primers for the tomato Actin gene (NM_001330119.1) and the *A. alternata* glyceraldehyde-3-phosphate dehydrogenase (*AaGAPDH*) gene (KJ717959.1) (Table S1).

### 2.5. Quantitative Real-Time Polymerase Chain Reaction (qRT-PCR)

The expression levels of *Alt-a1*, *TEF1a,* and *Tub2* were analyzed by qRT-PCR using SYBR Green I dye and a real-time PCR kit (Evrogen, Moscow, Russia) using two internal controls (*AaActin* and *AaGAPDH*). The expression was calculated by the 2^−∆∆CT^ method [21]. All gene identification numbers and used primers are listed in Table S1. qRT-PCR data shown were obtained from at least three experiments and are averages of 6 technical replicates for each experiment.

To quantify *A. alternata* DNA and assess the pathogen’s development in plant tissues, qRT-PCR was performed. Specific primers for *A. alternata* target genes (*Alt-a1*, *EF-1α*, *ITS*, *GAPDH*), as well as tomato housekeeping genes (*SlUbi*, *SlAct*), were developed specifically for RT-PCR (Table S1). DNA isolated from tomato leaves affected by Alternaria leaf spot was used as internal standards to construct a calibration curve. The resulting amplification levels for *A. alternata* sequences were divided by the values obtained for the *SlUbi* and *SlAct* genes from the corresponding samples. Sixteen technical replicates (eight qRT-PCR reactions normalized to the *SlUbi* (Solyc07g064130.1) gene region and eight qRT-PCR reactions to *SlAct* (Solyc04g011500.2)) were used to quantify the amplification of the studied sequences.

### 2.6. Chlorophyll Extraction and Quantification

Extraction and quantification of chlorophyll in leaves were performed according to a modified protocol [22]. Briefly, leaf samples were lyophilized to eliminate the effect of leaf moisture. Amounts of dried and crushed leaves (0.5 g) were transferred to a test tube and 10 mL of 80% *v*/*v* acetone were added. The samples were incubated in the dark at room temperature for 6 h, shaking the tube once an hour during the extraction period, until the crushed leaves became bleached. Absorbance at 646 nm and 663 nm was determined using a Spectrostar nano spectrophotometer (BMG Labtech, Ortenberg, Germany). Three biological replicates were used for each sample. The chlorophyll a and b contents were calculated using the following formulas: *C_A_* = (12.25 × *A*_663_ − 2.79 × *A*_646_) × *V*/(1000 × *W*); *C_B_* = (21.50 × *A*_646_ − 5.10 × *A*_663_) × *V*/(1000 × *W*), where *V* is the volume of the extraction solution and *W* is the weight of the sample in grams.

### 2.7. Statistical Analysis

The data, presented as mean ± standard error (SE), were subjected to a one-way analysis of variance (ANOVA) followed by Tukey’s pairwise comparison test. The 0.05 level was selected as the point of minimal statistical significance. For each type of analysis, at least three independent experiments were performed, each with several technical replicates.

## 3. Results

### 3.1. In Vitro Evaluation of Exogenous dsRNA Efficacy

**Figure 1:**
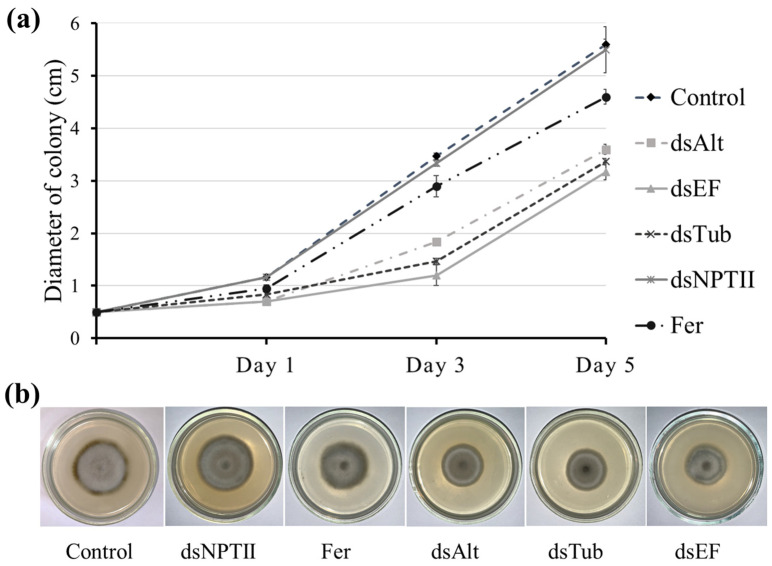
*Alternaria alternata* growth over time after application of water (Control); specific Alt-a1, TEF1a, and Tub2 dsRNA (dsAlt, dsEF and dsTub, respectively); nonspecific NPTII-dsRNA (dsNPTII) and the 0.2% solution of KS-carbendazim (Fer). (**a**) Colony diameter (cm) of fungi growing on potato dextrose agar (PDA) on days 1, 3 and 5 after treatment. (**b**) Examples of fungal development in Petri dishes on day 5 after treatment. Data are presented as mean ± standard error (SE).

Following the production of specific dsRNAs, their ability to inhibit the growth of *A. alternata* was assessed under in vitro conditions on Petri dishes. As a negative control, colonies were treated with 1 mL of sterile water. To compare the efficacy of the tested dsRNAs with a traditional plant protection approach, 1 mL of a 0.2% carbendazim fungicide solution was used. Carbendazim is a systemic, broad-spectrum agent from the benzimidazole class, commonly applied to control EB on tomatoes [23]. To confirm the specificity of the RNA interference effect, an additional control was included using 100 μg of dsRNA complementary to the *NPTII* gene, which is absent from the genomes of both the fungus and the tomato plant. It was shown that *A. alternata* colonies subjected to a single treatment with 100 μg of Tub2-, TEF1a-, or Alt-a1-dsRNA demonstrated a significant reduction in growth rate over the 5-day experiment compared to colonies treated with water, NPTII-dsRNA, or the fungicide (Figure 1a,b).

### 3.2. Protective Effect of Exogenous dsRNA Against A. alternata in Tomato Plants

**Figure 2:**
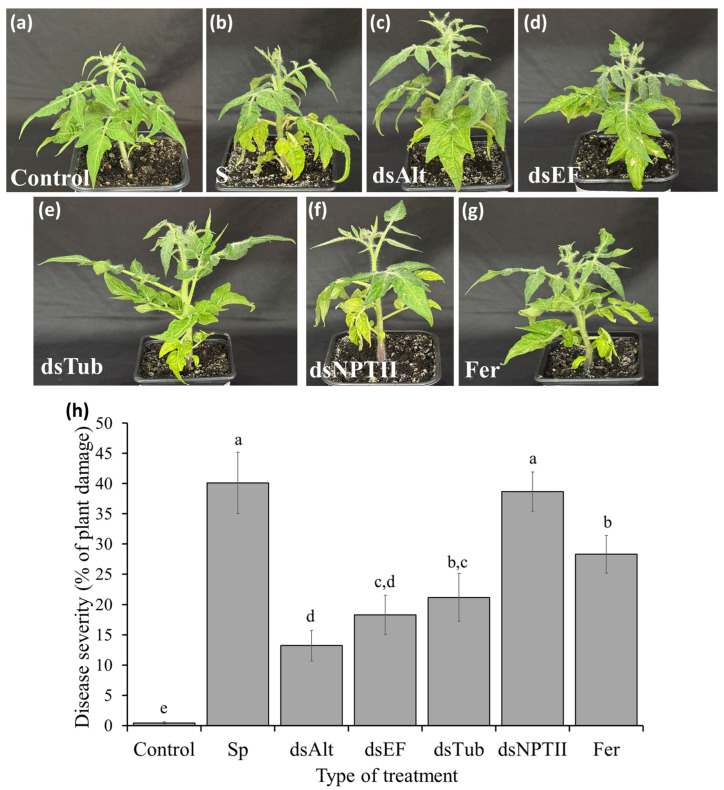
Tomato plant damage after inoculation with (**a**) water; (**b**) *A. alternata* spores; (**c**) *A. alternata* spores and Alt-a1-dsRNA; (**d**) *A. alternata* spores and TEF1a-dsRNA; (**e**) *A. alternata* spores and Tub2-dsRNA; (**f**) *A. alternata* spores and NPTII-dsRNA; (**g**) *A. alternata* spores and 0.2% solution of KS-carbendazim. (**h**) Percentage of affected area of tomato plants. Control—plants treated with 1 mL water; Sp—plants inoculated with *A. alternata* spores; dsAlt—plants inoculated with spores together with Alt-a1-dsRNA (100 μg); dsEF—plants inoculated with spores together with TEF1a-dsRNA (100 μg); dsTub—plants inoculated with spores together with Tub2-dsRNA (100 μg); dsNPTII—plants inoculated with spores together with NPTII-dsRNA (100 μg); Fer—plants inoculated with spores together with 1 mL of a 0.2% solution of the fungicide Ferazim (carbendazim 500 g/L). Quantification of the size of lesions caused by *A. alternata* was assessed using Fiji software. Data are presented as mean ± standard error (SE). Different letters above the bars indicate significant differences determined using one-way analysis of variance (ANOVA) with Tukey’s pairwise comparisons (*p* ≤ 0.05).

To assess the fungicidal potential of specific Tub2-, TEF1a-, and Alt-a1-dsRNA, a series of experiments was conducted on tomato plants. It was found that co-application of *A. alternata* spores with the target-specific dsRNAs led to a significant suppression of EB symptoms (Figure 2). The highest efficacy was demonstrated by the Alt-a1-dsRNA treatment, where the affected area of tomato leaves was reduced by 3 times compared to control plants inoculated with pathogen spores only (Figure 2b,h). The application of Tub2- and TEF1a-dsRNA also resulted in a significant reduction in leaf damage, by 1.8 and 2.2 times, respectively, compared to the control (Figure 2c,d,h). The use of NPTII-dsRNA did not have a significant effect on disease development. This indicates the specificity of the dsRNA action on the target gene sequences of the pathogen. Notably, the application of 0.2% solution of KS-carbendazim reduced the affected leaf area by only 1.4 times, which is lower than the efficacy achieved with all three specific dsRNA constructs (Figure 2g,h).

### 3.3. Chlorophyll Content

Physiological reactions and the ability of plants to withstand stress significantly affect the chlorophyll content [24]. Therefore, we next assessed the chlorophyll-a and chlorophyll-b content in tomato leaves after inoculation with *A. alternata* spores both alone and in combination with Tub2-dsRNA, TEF1a-dsRNA, Alt-a1-dsRNA, NPTII-dsRNA and 2% solution of the fungicide Ferazim (carbendazim 500 g/L).

**Figure 3:**
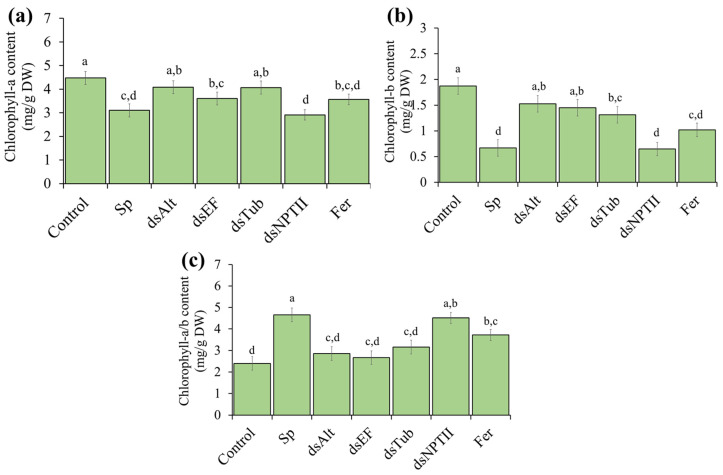
(**a**) Chlorophyll-a and (**b**) Chlorophyll-b content in leaves of tomato leaves; (**c**) Chlorophyll a/b ratio in tomato leaves. Control—plants treated with 1 mL water; Sp—plants inoculated with *A. alternata* spores; dsAlt—plants inoculated with spores together with Alt-a1-dsRNA (100 μg); dsEF—plants inoculated with spores together with TEF1a-dsRNA (100 μg); dsTub—plants inoculated with spores together with Tub2-dsRNA (100 μg); dsNPTII—plants inoculated with spores together with NPTII-dsRNA (100 μg); Fer—plants inoculated with spores together with 1 mL of a 0.2% solution of KS-carbendazim. Data are presented as mean ± standard error (SE). The means in each column followed by the same letter did not differ when using one-way analysis of variance (ANOVA) with Tukey’s pairwise comparisons (*p* ≤ 0.05).

Both chlorophyll-a and chlorophyll-b content were significantly reduced after inoculation with spores and after treatment with NPTII-dsRNA (Figure 3a). However, with the use of specific Tub2-dsRNA, TEF1a-dsRNA and Alt-a1-dsRNA the reduction in chlorophyll content was less pronounced.

The chlorophyll a/b ratio in tomato leaves increased significantly after spore inoculation and NPTII-dsRNA treatment compared to the control (Figure 3c). In contrast, the chlorophyll a/b ratio did not change significantly after Tub2-, TEF1a-, and Alt-a1-dsRNAs treatment (Figure 3c). Alternaria infection is known to cause damage to thylakoid membranes [25]. This destruction may occur unevenly. It can be speculated that chlorophyll b, associated with more vulnerable light-harvesting complexes, is destroyed faster than chlorophyll a in the reaction centers.

### 3.4. Effect of Double-Stranded RNAs on the Silencing Efficiency of Target Genes in A. alternata

**Figure 4:**
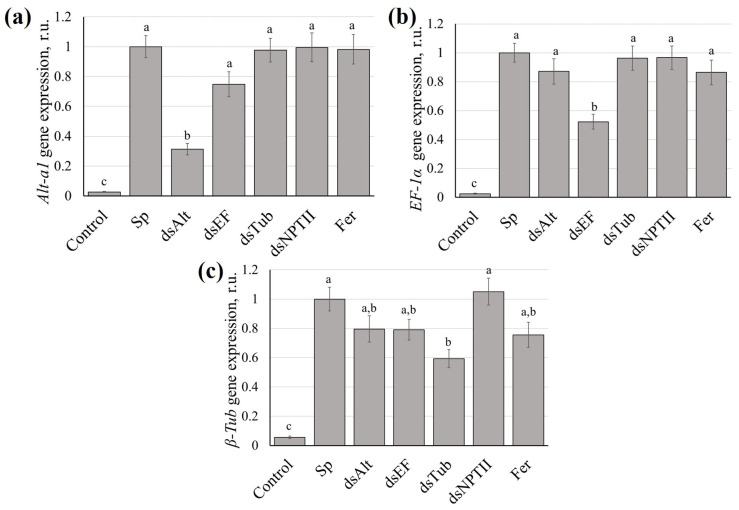
Changes in the expression of the (**a**) *Alt-a1*, (**b**) *TEF1a*, (**c**) *Tub2* genes of *Alternaria alternata.* Control—plants treated with 1 mL water; Sp—plants inoculated with *A. alternata* spores; dsAlt—plants inoculated with spores together with Alt-a1-dsRNA (100 μg); dsEF—plants inoculated with spores together with TEF1a-dsRNA (100 μg); dsTub—plants inoculated with spores together with Tub2-dsRNA (100 μg); dsNPTII—plants inoculated with spores together with NPTII-dsRNA (100 μg); Fer—plants inoculated with spores together with 1 mL of a 0.2% solution of KS-carbendazim. Expression analysis was assessed on day 7 post-inoculation (7 dpi). Data are presented as mean ± standard error (SE). The means in each column followed by the same letter did not differ when using one-way analysis of variance (ANOVA) with Tukey’s pairwise comparisons (*p* ≤ 0.05).

We assessed the effect of exogenous application of synthetic Tub2-dsRNA, TEF1a-dsRNA, Alt-a1-dsRNA, and NPTII-dsRNA onto the surface of 8-week-old tomato plants inoculated with *A. alternata* on the mRNA levels of the fungal *Alt-a1*, *EF-1α*, and *β-Tub* genes. Transcript levels were measured by RT-qPCR at 7 days post-inoculation (7 dpi). Treatment with Alt-a1-dsRNA resulted in a statistically significant 3.2-fold reduction in *Alt-a1* expression compared to the control (Figure 4a). TEF1a-dsRNA treatment led to a 1.9-fold suppression of *TEF1a* expression (Figure 4b). Tub2-dsRNA treatment caused a 1.7-fold decrease in *β-Tub* expression (Figure 4c). Treatment with NPTII-dsRNA or 0.2% solution of the fungicide, used as negative controls, did not induce significant changes in target transcript levels, confirming the specificity of dsRNA action (Figure 4).

### 3.5. Relative Quantitative Determination of A. alternata DNA

Valsesi et al. [21] developed a multiplex real-time polymerase chain reaction (real-time PCR) assay utilizing TaqMan fluorescent probes for the relative quantification of *Plasmopara viticola* DNA directly from leaf tissue of *Vitis vinifera* [26]. Subsequently, Pavon and colleagues developed a real-time PCR method for the specific detection of *Alternaria* spp. in food products. This approach was based on primers targeting the internal transcribed spacers ITS1 and ITS2 of the ribosomal RNA (rRNA) gene [27].

**Figure 5:**
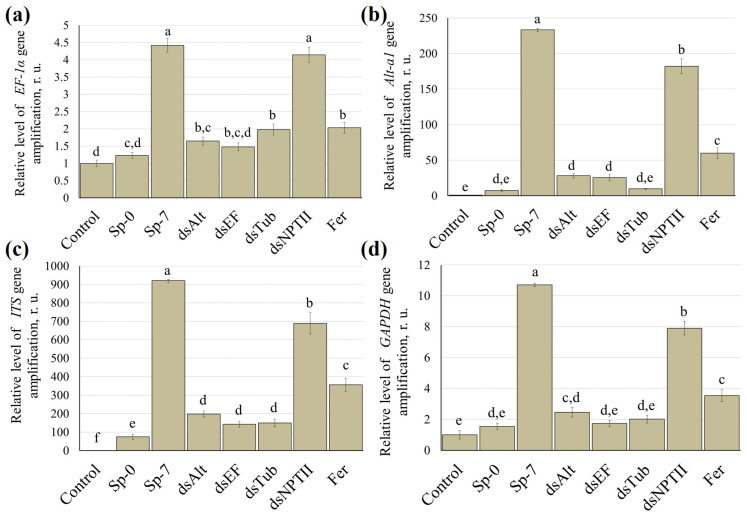
Relative amplification level of *Alternaria alternata* DNA of different genetic markers: (**a**) translation elongation factor 1-α (*EF-1α*) gene; (**b**) allergen Alt a 1 (*Alt-a1*) gene; (**c**) internal transcribed spacer (*ITS*); (**d**) glyceraldehyde-3-phosphate dehydrogenase (*GAPDH*) gene. Control—plants treated with 1 mL water; Sp-0—plants inoculated with *A. alternata* spores at 1 hpi; Sp-7—plants inoculated with *A. alternata* spores at 7 dpi; dsAlt—plants inoculated with spores together with Alt-a1-dsRNA (100 μg); dsEF—plants inoculated with spores together with TEF1a-dsRNA (100 μg); dsTub—plants inoculated with spores together with Tub2-dsRNA (100 μg); dsNPTII—plants inoculated with spores together with NPTII-dsRNA (100 μg); Fer—plants inoculated with spores together with 1 mL of a 0.2% solution of KS-carbendazim. Analysis was assessed on day 7 post-inoculation (7 dpi). Data are presented as mean ± standard error (SE). The means in each column followed by the same letter did not differ when using one-way analysis of variance (ANOVA) with Tukey’s pairwise comparisons (*p* ≤ 0.05).

In this study, to assess the efficacy of dsRNA on the development of *A. alternata* on tomatoes, quantitative DNA analysis was performed using real-time PCR with primers listed in Table S1. The relative amplification level was calculated as the ratio of fungal DNA to plant DNA in each sample. For the detection of *A. alternata*, the *Alt-a1*, *EF-1α*, *ITS* and *GAPDH* genes were used as targets. According to the real-time PCR data, the relative amplicon levels of all target genes were significantly higher in spore-treated plants at 7 days post-inoculation with *A. alternata* spores compared to control plants treated with water (Figure 5). DNA was also extracted from plants 1 h after inoculation with *A. alternata* spores to assess the baseline amplification level of fungal DNA amplification immediately after spore application, before active growth began. This control allowed us to confirm that the detected signal was indeed due to the introduced *A. alternata* spores and not to contamination of the samples. It was shown that a statistically significant difference between 1 hpi and the control was only observed when using primers targeting the *ITS* gene (Figure 5c).

The maximum accumulation level of pathogen DNA was observed for the *ITS* gene, indicating its higher sensitivity (Figure 5c). In contrast, the lowest relative amplification values were characteristic of the *GAPDH* and *TEF1a* genes (Figure 5a,d). The application of Alt-a1-dsRNA, TEF1a-dsRNA, and Tub2-dsRNA together with spore inoculation led to a significant reduction in the amplicon levels of all target genes of the pathogenic fungus, ranging from 9- to 27-fold, compared to plants inoculated with spores only (Figure 5a–d). The use of a fungicide also reduced amplicon levels at 7 days post-inoculation, but to a lesser extent, from 2.1- to 3.8-fold, compared to control plants treated with *A. alternata* spores only (Figure 5a–d). Interestingly, treatment with NPTII-dsRNA caused a slight reduction in amplicon levels; however, these differences were statistically significant only for the *Alt-a1*, *ITS* and *GAPDH* genes (Figure 5b–d).

## 4. Discussion

In this study, we demonstrated for the first time the successful use of exogenous dsRNA as a highly specific and effective tool for the suppression of the phytopathogenic fungus *A. alternata* on tomato plants. Using the SIGS approach, we showed that a single co-application of *A. alternata* spores with dsRNA targeting the *Alt-a1*, *EF-1α*, or *β-Tub* genes significantly reduced EB symptoms, reduced the amount of fungal DNA in plant tissues, and partially preserved photosynthetic pigment content. These results provide compelling evidence that SIGS may be a viable alternative or complement to traditional chemical fungicides for the control of Alternaria leaf spot.

Among the three tested constructs, Alt-a1-dsRNA consistently demonstrated the highest protective effect. It reduced *Alt-a1* transcript levels by 3.2-fold (Figure 4a) and reduced pathogen DNA more effectively than dsRNAs targeting *TEF1a* or *β-Tub* (Figure 5). The *Alt-a1* gene encodes the major allergen and a well-established virulence factor of *A. alternata* [4,28]. This gene is directly involved in the ability of the pathogen to colonize host tissues and evade plant defense responses [28]. Silencing a virulence-related gene may impose a greater fitness constraint on the fungus than silencing housekeeping genes such as *TEF1a* or *β-Tub* [29]. Housekeeping genes are essential for survival, but fungi may have compensatory mechanisms or duplicated genes that partially counteract the effect of their suppression [30]. At the same time, the loss of a specific virulence factor can directly disrupt the infection process. Our results are consistent with previous reports that inhibition of virulence factors often provides more effective disease control than targeting general metabolic genes [30,31,32]. It was shown that suppression of the *ACTT2* or *ACTTS2* genes of *A. alternata*, encoding hydrolase, by transforming the fungus with a plasmid construct expressing hairpin RNAs, resulted in a complete loss of ACTT2 and ACTTS2 transcripts, and as a consequence, the production of ACT toxin and virulence [31,32]. Therefore, we suggest that *Alt-a1* represents a promising target for EB treatment by SIGS.

One of the interesting results of our study was the superiority of the use of Alt-a1-dsRNA compared to the fungicide based on carbendazim, which acts by inhibiting β-tubulin [33]. The use of a fungicide resulted in a decrease in the area of affected leaves by only 1.4 times and reduced the amount of pathogen DNA from 2.1 to 3.8 times, treatment with Alt-a1-dsRNA led to a three-fold reduction in the area of damage to tomato plants and up to a 27-fold decrease in the relative level of amplification of the *ITS* marker of *A. alternata* (Figure 5). However, interestingly, the results of using Tub2-dsRNA and fungicide were comparable in a number of ways, including a decrease in the area of leaf damage, chlorophyll content and expression of the *Tub2* genes of *A. alternata* (Figure 4c). This similarity may be that, despite different mechanisms of action, both approaches ultimately affect the microtubular function of the pathogen: carbendazim directly disrupts the polymerization of β-tubulin, while Tub2-dsRNA reduces the expression level of the corresponding gene at the post-transcriptional level [34].

The specificity of the observed RNA interference effect was confirmed by the lack of significant disease suppression with NPTII-dsRNA, which targets the neomycin phosphotransferase II gene, the sequence of which is absent from both the *A. alternata* and tomato genomes. A small but statistically significant decrease in the amplicon levels of some markers, namely *Alt-a1*, *ITS*, and *GAPDH*, was observed with NPTII-dsRNA treatment (Figure 5). This effect could be explained by a nonspecific immune response in the fungus, such as activation of the RNA interference mechanism by any long dsRNA, or by a very low level of sequence-independent off-target effects. However, the magnitude of this effect was incomparably lower than that observed with specific dsRNA.

The efficacy of SIGS observed in this study is consistent with previous studies using exogenous dsRNA to inhibit the development of fungal pathogens. For example, dsRNA targeting the *CYP51* and *EF2* genes successfully suppressed *F. graminearum* and *B. cinerea* in barley, Arabidopsis and grapes [14,15,16]. Similarly, spraying with dsRNA against the *TEF1a* gene of *P. infestans* significantly reduced the growth rate, sporulation, disease severity, and decreased the expression of pathogen target genes on potato [17]. In another study, Gu et al. used dsRNA targeting the *Faβ2Tub* genes of *B. cinerea* and *Colletotrichum truncatum*, which led to inhibition of fungal growth and weaker disease symptoms at 4 dpi [35]. However, to our knowledge, our study is one of the first detailed reports demonstrating the effectiveness of SIGS against *Alternaria* on tomato using multiple independent target genes and a comprehensive set of evaluation criteria. Thus, this study expands the list of phytopathogenic fungi amenable to control by SIGS.

Despite the promising results, we acknowledge several limitations of this study. First, all experiments were conducted under controlled conditions. Field conditions include variable temperatures, humidity, UV radiation, and precipitation, which may degrade dsRNA or reduce its uptake by the pathogen [36,37]. Second, we applied a single dose of dsRNA (100 μg per plant) simultaneously with fungal inoculation. Long-term protection and the need for repeated treatments were not assessed in a longer experiment. Third, the mechanism by which exogenous dsRNA applied to the leaf surface penetrates *A. alternata* hyphae remains unclear [38]. Possible routes include direct uptake through the fungal cell wall (via endocytosis or specific transporters), uptake from apoplastic fluid, or even uptake through damaged plant tissue [39,40]. Elucidating these mechanisms will be important for optimizing dsRNA delivery and stability.

Thus, our study clearly demonstrates that exogenous dsRNA targeting the *Alt-a1*, *TEF1a* and *β-Tub* genes of the fungus *A. alternata* effectively suppresses EB development in tomato plants. The *Alt-a1* gene emerged as the most promising target. These results support the further development of SIGS as an environmentally friendly, highly specific, and effective strategy for controlling Alternaria blight and, potentially, other fungal diseases in agriculture. With continued research into dsRNA stabilization, delivery, and field effectiveness, SIGS has the potential to become an effective next-generation plant protection technology.

## Data Availability

The data presented in this study are available within the article and Supplementary Materials.

## Supplementary Materials

The following supporting information can be downloaded at: https://www.mdpi.com/article/10.3390/jof12050373/s1; Table S1: Primers used in the work.
