# Supplementary material for: Targeted Suppression of the Tomato Pathogen Alternaria alternata via Exogenous Application of Double-Stranded RNA
Source: J Fungi (Basel). 2026 May 18;12(5):373. doi: 10.3390/jof12050373 (PMC13208992; doi:10.3390/jof12050373)
Supplement: Supplementary file 1 [file jof-12-00373-s001.zip › jof-4292380-supplementary.pdf]

Table S1 Primers used in the work.

Gene name (ID number)	Primer name	Primers, 5′-3′
Primers for cloning cDNA coding sequences, 5′-3′		
Alt a1 (MW387003.1)	Alt-for	ATGCAGTTCACCACCATCGC
	Alt-rev	ACGAGGGTGAYGTAGGCGTC
Elongation factor-1a (XM_018536149.1)	Al-EF-F	CGGTACTGGTGAGTTCGAGG
	Al-EF-R	CACCGTGCCAATACCACCAA
beta-tubulin (KY814630.1)	TUB2-T1-F	ATGCGTGAGATTGTTCACCT
	TUB2-Bt2b-R	ACCCTCAGTGTAGTGACCCTTGGC
NPTII (AY818371)	NPTII-nac	ATGTGGATTGAACAAGATGG
	NPTII-kon	TCAGAAGAACTCGTCAAGAA
**Specific primers for dsRNA design, 5′-3′**		
Alt a1 (MW387003.1)	Alt-dsRNA-F	TAATACGACTCACTATAGGGAGA-ATGCAGTTCACCACCATCGC
	Alt-dsRNA-R	TAATACGACTCACTATAGGGAGA-GAATCTGCGAAGTAATCGAAG
Elongation factor-1a (XM_018536149.1)	EF-dsRNA-F	TAATACGACTCACTATAGGGAGA-CGGTACTGGTGAGTTCGAGG
	EF-dsRNA-R	TAATACGACTCACTATAGGGAGA-CACCGTGCCAATACCACCAA
beta-tubulin (KY814630.1)	Tub-dsRNA-F	TAATACGACTCACTATAGGGAGA-ATGCGTGAGATTGTTCACCT
	Tub-dsRNA-R	TAATACGACTCACTATAGGGAGA-GGCTCGAGATCGACGAGGAC
NPTII (AY818371)	npt-T71-s	TAATACGACTCACTATAGGGAGA-ATGTGGATTGAACAAGATGGATTG
	npt-T72-a	TAATACGACTCACTATAGGGAGA-TCCACCATGATATTCGGCAAGCAG
**Primers for real-time PCR, 5′-3′**		
Alt a1 (MW387003.1)	Alt-real-S	TTTCGACAGCGACCGCAACG
	Alt-real-A	GCGCGGCAGTAGTTGGGAAG
Elongation factor-1a (XM_018536149.1)	Aa-EF-real-S2	CCGGTATCATCAAGGCCGGT
	Aa-EF-real-A2	GTGGTGCATCTCGACGGACT
beta-tubulin (KY814630.1)	Aa-Tub-real-S2	GCTGTTCCGCCCTGACAAC
	Aa-Tub-real-A2	CCAGTTGTTACCAGCACCAGAC
SlActin (Solyc04g011500.2)	SlAct-realS	GAAATAGCATAAGATGGCAGACG
	SlAct-realA	ATACCCACCATCACACCAGTAT
SlUBI (Solyc07g064130.1)	SlUBI-realS	GGACGGACGTACTCTAGCTGAT
	SlUBI-realA	AGCTTTCGACCTCAAGGGTA
AaActin (MT479097.1)	Aa-Act-real-S2	GGCCGACCGCGTCAC
	Aa-Act-real-A2	GAGTCCTTCTGGCCCATACCA
AaGAPDH (KJ717959.1)	AaGAPDH-real-S2	CATGTTCGTCATGGGCGTCA
	AaGAPDH-real-A2	AGAGGCGTTGGAGAGAACCT
**Primers for cDNA check-up on DNA contamination, 5′-3′**		
SlActin (Solyc04g011500.2)	SlAct-OT-s	ATGGCAGACGGAGAGGAT
	SlAct-OT-a	TTCACGATTAGCCTTTGG GT
AaGAPDH (KJ717959.1)	AaGAPDH-OT-S	CGTAAACGACCCCTTCATCG
	AaGAPDH-OT-A	AGAGGCGTTGGAGAGAACCT
